# Clinical, Cognitive, and MRI Profiles in a Memory Clinic Cohort with Negative Versus Positive PET Amyloid Scans

**DOI:** 10.1002/alz70857_103158

**Published:** 2025-12-25

**Authors:** Hessah A Alotibi, Juan‐Camilo Vargas‐González, Alia Alokley, Martin Ingelsson, David F. Tang‐Wai, Carmela Tartaglia

**Affiliations:** ^1^ Memory Clinic, Toronto Western Hospital, University Health Network, Toronto, ON, Canada; ^2^ University Health Network, Toronto, ON, Canada; ^3^ Department of Public Health and Caring Sciences, Uppsala University, Sweden, Sweden; ^4^ Toronto Dementia Research Alliance, Toronto, ON, Canada; ^5^ Temerty Faculty of Medicine, University of Toronto, Toronto, ON, Canada; ^6^ Krembil Brain Institute, University Health Network (UHN), Toronto, ON, Canada; ^7^ Tanz Centre for Research in Neurodegenerative Diseases, University of Toronto, Toronto, ON, Canada; ^8^ Toronto Dementia Research Alliance (TDRA), Toronto, ON, Canada

## Abstract

**Background:**

Patients with memory concerns but negative Positron Emission Tomography (PET) amyloid scans pose significant diagnostic challenges, requiring differentiation between non–Alzheimer's disease (AD) pathologies. Limbic‐predominant Age‐related TDP‐43 Encephalopathy (LATE) has emerged as a notable alternative diagnosis, characterized by impaired delayed recall, preserved verbal fluency, and medial temporal atrophy^12^. Understanding the cognitive and MRI features that distinguish amyloid‐negative from amyloid‐positive patients is essential for refining diagnostic accuracy

**Method:**

This descriptive study included 56 patients with cognitive complaint (28 amyloid‐PET‐ negative, 28 amyloid ‐PET‐positive) who were evaluated between 2023 and 2025 at the UHN Memory Clinic and had a positron emission tomography (PET), MRI and Toronto Cognitive Assessment (ToRCA). Demographic, cognitive, amyloid PET scan and MRI data were compared, with medians and interquartile ranges (IQR) reported for non‐normally distributed variables.

**Result:**

Both groups had similar age (70.0 [65.5–76.8] vs. 74.5 [66.0–78.0], *p* = 0.768) and sex distribution (57.1% male, *p* = 1.000). Amnestic MCI was more frequent in the amyloid‐negative group (89.3% vs. 57.1%, *p* = 0.016), while dementia was more common in the amyloid‐positive group. Amyloid‐negative patients showed better delayed recall (3.00 [2.75–5.00] vs. 1.00 [0.00–3.25], *p* = 0.008), delayed recognition (19.00 [16.00–20.00] vs. 17.00 [12.00–18.00], *p* = 0.006), and visuospatial memory (Benson Figure Delayed Recall: 10.00 [8.00–12.00] vs. 8.00 [3.00–10.25], *p* = 0.021) than the amyloid‐positive group. Semantic fluency (animals) was also higher (16.00 [12.00–18.25] vs. 13.00 [7.75–15.25], *p* = 0.006) in the amyloid‐negative patients, while lexical fluency (F words) was comparable (*p* = 0.134). There were no significant differences in global cortical atrophy or moderate‐to‐severe atrophy of the medial temporal lobes.

**Conclusion:**

This descriptive study suggests that amyloid‐negative patients are more likely to present with aMCI than dementia, and exhibit milder memory deficits than amyloid‐positive patients, aligning with reports that LATE may be less severe than AD. Though referral bias may partly explain the higher proportion of aMCI in the amyloid‐negative group, these findings underscore the need to consider non‐AD pathologies in patients with memory complaints and negative amyloid scans. Future research may elucidate distinct clinical signatures to improve diagnostic workflows in memory clinics.

